# Extended Criteria Donor Use in Heart Transplantation: A Promising Strategy to Expand the Donor Pool

**DOI:** 10.3390/jcm15051980

**Published:** 2026-03-05

**Authors:** Giuseppe Fischetti, Lorenzo Giovannico, Domenico Parigino, Luca Savino, Federica Mazzone, Claudia Leo, Giuseppe Cristiano, Martina Macella, Paola De Santis, Federico Scalese, Eduardo Urgesi, Nicola Di Bari, Concetta Losito, Aldo Domenico Milano, Massimo Padalino, Massimiliano Carrozzini, Tomaso Bottio

**Affiliations:** 1 Cardiac Surgery Unit, Department of Precision and Regenerative Medicine and Ionian Area (DiMePRe-J), University of Bari Medical School, Piazza Giulio Cesare 11, 70100 Bari, Italy; giuseppefischetti23@gmail.com (G.F.); lorenzo.giovannico92@gmail.com (L.G.); domenicoparigino@gmail.com (D.P.); lucasavino2905@gmail.com (L.S.); claudialeo2810@gmail.com (C.L.); giuseppecristiano88@gmail.com (G.C.); martina.macella145@gmail.com (M.M.); paoladesantis0@gmail.com (P.D.S.); federicoscalese@gmail.com (F.S.); dibari.nicola77@gmail.com (N.D.B.); dottclosito@libero.it (C.L.); aldo.milano@uniba.it (A.D.M.); massimo.padalino@uniba.it (M.P.);; 2 Department of Integrated Activities (DAI) Cardiothoracic, University Cardiology, University of Bari School of Medicine, Piazza Giulio Cesare 11, 70100 Bari, Italy; federica.mazzone95@gmail.com (F.M.); eduardourgesi@gmail.com (E.U.)

**Keywords:** heart transplantation, shortage organ, donor, recipient, ECMO

## Abstract

**Background**: To address organ shortage and reduce waitlist mortality, the use of extended criteria donors (ECDs) in heart transplantation is increasing. **Methods**: We retrospectively analysed outcomes in 236 heart transplant recipients: 140 received standard donor (SD) hearts and 96 received ECD hearts. **Results**: No significant differences were found in early or mid-term survival between the SD and ECD groups with a 30-day mortality rates of 13% vs. 10% (*p* = 0.662) and estimated 1-year survival of 75% (95% CI: 62.3–78.3%) and 71% (95% CI: 55.3–76.2%) (*p* = 0.556), respectively. Mechanical ventilation prior to transplant (*p* < 0.001), ischemic time (*p* = 0.022), peripheral vascular disease (*p* = 0.011), and chronic obstructive pulmonary disease (*p* = 0.022) were the only independent predictors of mortality. **Conclusions**: In our cohort, heart transplantation using ECD was not associated with increased early or mid-term adverse events. This approach may help expand the donor pool without compromising post-transplant outcomes.

## 1. Introduction

Currently, heart transplantation (HTx) represents the gold-standard surgical approach in the treatment of refractory heart failure (HF), which is often associated with significant functional limitations and a high mortality rate [1]. In Italy, patients typically wait between three and five years to undergo heart transplantation, depending on the geographical region and baseline clinical status. Concerning mortality on the waiting list, large-scale data in Italy remain limited. However, at the European level, it is estimated that about 7% of patients on the HTx waitlist die each year before receiving their organ [2]. As a result of the increasing demand for donor grafts, the criteria for acceptable organs are being challenged. Donors who were once viewed as “marginal” are being increasingly accepted for transplantation. Evidence-based donor selection and management guidelines may consolidate our current knowledge in order to maximize the use of available donor hearts for transplantation [3,4]. Despite the increasing use of ventricular assist devices, the selection and use of donors with extended criteria may represent a potential solution to address the growing demand for cardiac organs [5]. This study presents one of the first Italian experiences with systematic use of extended criteria donors (ECDs), including donors over 60 years and broader institutional selection criteria than those commonly applied in other European cohorts. The aim of this study was to compare early and mid-term outcomes among recipients of standard donor hearts (SD) and recipients of extended criteria donors (ECD).

## 2. Materials and Methods

### 2.1. Study Population

This is a retrospective observational study based on prospectively collected data from all patients who received HTx at our institution between March 2022 and September 2025. This period corresponds to the introduction and systematic use of ECDs at our institution. Patient anonymity was respected at the time of admission. This study was conducted in accordance with the ethical principles outlined in the Declaration of Helsinki (1975, as revised 2013). The study protocol was reviewed and approved by the Institutional Ethics Committee of the University of Bari (Approval No. 7835, 24 April 2024). Informed consent for participation and use of clinical data for research purposes was obtained from all individual patients prior to inclusion in the study.

### 2.2. Outcomes and Definition of Extended Criteria Donor

The primary outcomes were 30-day, in-hospital, and follow-up mortality. Secondary outcomes included the need for veno-arterial extracorporeal membrane oxygenation (VA-ECMO) and the rate of adverse procedural events. ECDs were defined according to one or more of the following characteristics: age > 60 years, documented significant coronary artery disease, left ventricular ejection fraction (EF) < 45%, presence of cardiac defects requiring surgical correction at the time of HTx, excluding simple atrial septal defect. During the study period, the heart transplant waiting list in our Organ Procurement Organization was structured as follows:

- Emergency Status 1 (National Emergency): Patient requiring short-term mechanical circulatory support; complicated total artificial heart; complicated biventricular EXCOR^®^ -VAD; LVAD carriers with at least one severe complication.
- Emergency Status 2 (Macro-area Emergency): Hospitalized patients not eligible for LVAD implant with IABP or micro-axial flow short-term support, continuous inotrope infusion for more than seven days, major arrhythmias not susceptible to ablation, complicated grown-up congenital heart disease, biventricular EXCOR^®^ -VAD carriers for >3 months, total artificial heart carriers for >3 months, L-VAD carriers with at least two minor complications;
- Non-hospitalized patients [6].

### 2.3. Statistical Analysis

Categorical data were expressed as frequencies and percentages, while continuous data were expressed as mean ± standard deviation or median and interquartile range (IQR), depending on the distribution. Comparisons between the two groups were performed using Student’s *t*-test for normally distributed continuous data and the Mann–Whitney U-test for non-normally distributed data. Differences in proportions were analyzed using the chi-square test or Fisher’s exact test, as appropriate. Survival analysis was performed using Kaplan–Meier curves, and differences in survival distributions were assessed with the log-rank test. To identify independent predictors of post-transplant mortality, a multivariate Cox regression analysis was conducted. All characteristics of interest were first analyzed independently, via univariate regression analysis. The final model was selected using a backward stepwise procedure including all clinically and statistically significant variables (*p* < 0.20) and guided by changes greater than 10 points in the Akaike Information Criterion (AIC). Results are reported as hazard ratios (HRs) with 95% confidence intervals (CIs). A value of *p* < 0.05 was considered statistically significant. All statistical analyses were performed using StataMP software, version 18 StataMP version 18 (StataCorp LLC, College Station, TX, USA).

## 3. Results

We included a total of 236 patients. Among these, 140 patients (60%) received a standard donor organ, while 96 patients (40%) received an extended criteria donor heart.

Recipients of ECD were significantly older compared to those who received SD [59 (51.5–64) vs. 64 (58–68) years, (*p* = 0.000)]. A higher proportion of patients in the SD group were on mechanical ventilation prior to HTx (*p* = 0.013) and required CRRT pre-HTx (*p* = 0.012). All the other baseline characteristics were comparable between the two groups (Table 1).

ECD were significantly older and had a higher prevalence of dyslipidemia, hypertension, and diabetes, compared to SD (all *p* < 0.05). These findings reflect a greater comorbidity burden among ECD (Table 2).

### 3.1. Post-Operative Outcomes

**Figure 1:**
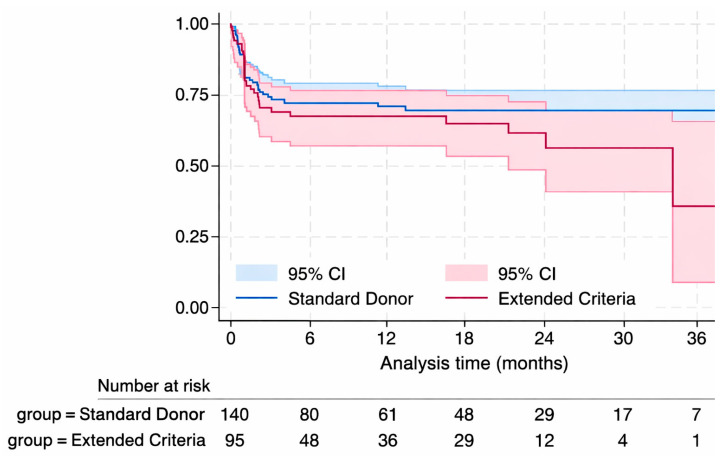
Kaplan–Meier survival curves for recipients of standard donor (SD, blue) and extended criteria donor (ECD, red) hearts. Shaded areas show 95% confidence intervals; numbers below indicate patients at risk over time.

Post-HTx outcomes were comparable between the two groups (Table 3). Kaplan–Meier survival analysis demonstrated no statistically significant difference in survival during follow-up between SD and ECD recipients (LogRank Test *p*-value = 0.285), with an estimated 1-year survival of 75% (95% CI: 62.3–78.3%) and 71% (95% CI: 55.3–76.2%) (*p* = 0.556), respectively (Figure 1).

### 3.2. Predictors of Mortality

Predictors of mortality were assessed in the entire cohort, including both SD and ECD recipients. Results of the univariate Cox regression analysis are shown in Table 4. Redo cardiac surgery (HR 1.69, *p* = 0.036), mechanical ventilation (MV) prior to HTx (HR 3.71, *p* < 0.001), continuous renal replacement (CRRT) before HTx (HR 2.62, *p* < 0.007), veno-arterial extracorporeal membrane oxygenation (VA- ECMO) before HTx (HR 2.59, *p* < 0.001), donor diabetes (HR 2.28, *p* = 0.003), and post-transplant complications such as graft rejection (HR 1.95, *p* = 0.016), need for VA-ECMO (HR 2.92, *p* < 0.001) or CRRT (HR 5.60, *p* < 0.001), and infection (HR 4.16, *p* < 0.001) were found to be strongly associated with mortality.

In our final multivariate Cox regression model (PH test *p* = 0.265; Harrel’s C: 72.65%), the strongest independent predictor of mortality was mechanical ventilation prior to HTx (HR 5.11, *p* < 0.001). Peripheral vascular disease (PVD) (HR 1.96, *p* = 0.011), chronic obstructive pulmonary disease (COPD) (HR 1.81, *p* = 0.022), donor diabetes (HR 2.29, *p* = 0.006), and ischemic time (HR 1.00, *p* = 0.020) also remained significant predictors of mortality. Conversely, extended donor criteria (*p* = 0.176) and recipient age (*p* = 0.176) were not independently associated with mortality (Table 5).

## 4. Discussion

In recent years, the number of patients on the heart transplant waiting list has steadily increased. There is a persistent imbalance between the growing number of heart transplant candidates and the limited availability of donor hearts in both the United States and Europe [7]. Advances in medical therapy for advanced heart failure have improved patient survival, consequently delaying the need for heart transplantation and allowing for more strategic donor heart allocation [8]. According to the Italian National Heart Transplant Database, approximately 10% of candidates die within the first year of being waitlisted. This substantial waitlist mortality reflects the persistent mismatch between organ supply and demand, supporting the rationale for the selective use of extended criteria donors (ECDs). Although associated with a potentially higher perioperative risk, ECD utilization effectively broadens the donor pool and contributes to a measurable reduction in waitlist mortality [9]. While LVADs are a valuable and durable therapy for advanced heart failure, not all patients are suitable candidates due to comorbidities and potential complications requiring careful selection. Complications such as late right heart failure, recurrent hospitalizations, driveline infections, and gastrointestinal bleeding remain major concerns after LVAD implantation, significantly impacting morbidity and survival [10]. At the same time, organ donation in Europe has undergone significant changes. United States heart donors are generally younger, with a mean age of approximately thirty years and fewer comorbidities, reflecting stricter donor selection criteria. In contrast, European heart donors are older and have more associated comorbidities [11]. The persistent imbalance between the number of transplant candidates and available donor hearts has led many centers to expand their acceptance criteria, with an increasing reliance on extended criteria donors to meet the growing demand for organ transplants. To address this imbalance and reduce waitlist mortality—which ranges from approximately 5% in Spain to over 10% in countries such as France and Germany, and around 8–10% in Italy—transplant centers are increasingly considering the use of hearts from extended criteria donors [12,13,14]. The use of extended criteria donors has been shown to reduce waitlist mortality without significantly compromising post-transplant outcomes, as demonstrated by Lietz et al. [15]. In our study, we compared outcomes between recipients of SD and ECDs. We found no significant differences between the two groups in either short-term or mid-term survival. A similar survival was also reported in the study by Bifulco et al. [16]. One-year survival in recipients of hearts from marginal donors was 70%, confirming the possibility of obtaining satisfactory outcomes even with ECD [17]. However, it is worth noting that overall mortality in our cohort is higher than that reported in major U.S. registries, where adult heart transplant recipients have demonstrated 1-year survival rates of approximately 90.6% and 5-year survival rates of 79.5% [18]. Our 1-year survival rate, however, is consistent with outcomes reported in European cohorts, including recent ISHLT registry data and Eurotransplant experience, where survival typically ranges between 70% and 80% among recipients with similar profiles [19,20]. Compared to U.S. recipients, who are typically younger, Italian heart transplant recipients in our cohort were older and more clinically complex, with higher rates of kidney dysfunction and elevated levels of bilirubin, likely contributing to the increased mortality. This reflects a global trend in heart transplantation, in which increasingly complex recipients are being considered for listing and transplant eligibility [21]. Our results identified pre-transplant mechanical ventilation as the strongest independent predictor of mortality (HR 5.11; 95% CI 2.70–9.69; *p* < 0.001). Peripheral vascular disease (HR 1.81; 95% CI 1.09–3.00; *p* = 0.011), chronic obstructive pulmonary disease (HR 1.81; 95% CI 1.09–3.00; *p* = 0.022), and ischemic time (HR 1.00; 95% CI 1.00–1.01; *p* = 0.022) were also independently associated with increased mortality, reflecting the complexity in our population. As reported by Miller et al., mechanical ventilation at the time of heart transplantation has been independently associated with significantly increased early and 1-year mortality, as shown in a UNOS-based analysis of over 60,000 patients [21]. Although overall mortality rates did not significantly differ between our study groups, a non-negligible proportion of deaths during follow-up was infection-related, with no statistically significant difference between the groups (41% vs. 45%; *p* = 0.595). This relatively high incidence warrants attention, especially given its potential contribution to early and late mortality. Previous studies have identified infections as one of the leading causes of death following heart transplantation, particularly in the early postoperative period when immunosuppression is most intense [22]. In our cohort characterized by moderate/severe pre-transplant renal function [median GFR: 60.0 (46.0–79.5) mL/min in SD and 55.5 (40.50–75.00) mL/min in ECD], a substantial proportion of patients required postoperative dialysis (CRRT in 45% of SD and 47% of ECD recipients). In line with the findings of Hong et al. [23], this significant incidence of post-transplant need for dialysis might have also contributed to the observed rates of mortality.

To overcome the shortage of donor organs, machine perfusion strategies have recently become crucial in modern transplant units and are considered promising tools to expand the donor pool. Results so far are encouraging, with no significant differences in the overall 2-year survival or incidence of severe primary graft dysfunction across various preservation modalities [24,25]. Similarly, donation after circulatory death (DCD) has demonstrated comparable or even superior survival outcomes compared to donation after brain death (DBD), with lower 1-year and 5-year mortality rates. These findings support DCD as a promising strategy to expand the heart donor pool [26]. However, while DCD, machine perfusion, and ECD represent innovative approaches to address the donor shortage, further evidence is still required to confirm their long-term effectiveness, clinical superiority, and systematic use.

The main limitations of this study include its retrospective design, although the data were prospectively collected, and its relatively short follow-up period. While follow-up is still ongoing, the present mid-term results are encouraging and suggest that the use of extended criteria donors (ECD) does not compromise transplant outcomes. Among patients with at least one year of follow-up, no cases of cardiac allograft vasculopathy (CAV) were observed. Longer-term follow-up of this cohort is ongoing, and an updated analysis including 2- and 3-year outcomes is planned to better assess late graft survival and complications. Given that single-center retrospective studies may inherently carry the risk of selection bias, the donor acceptance process in our program was based on predefined institutional criteria, including donor age, comorbidities, and cardiac function assessment.

Moreover, the absence of larger case series in the literature reflects the relatively recent adoption of extended criteria donors. As a result, albeit small, our cohort represents a valuable addition to the existing literature. The present study supports the clinical viability of using extended criteria donor (ECD) hearts in selected recipients, without a significant increase in early or long-term mortality. Despite the higher risk profile of both donors and recipients, outcomes were comparable to those observed with standard donors. Infection remains a major cause of morbidity, particularly in more complex cases, highlighting the need for tailored perioperative management. Overall, both analyses highlight the critical impact of recipient pre-transplant status, comorbidities, and donor diabetes, while post-transplant complications markedly worsen prognosis. These findings underscore the importance of individualized donor–recipient matching and the need for ongoing refinement of selection criteria to address the organ shortage safely.

## 5. Conclusions

Based on our findings, the use of ECD did not significantly impact early and mid-term outcomes of heart transplantation. In the context of the growing disparity between available donors and patients awaiting transplantation, ECD utilization may represent a valuable strategy to expand the donor pool and reduce waitlist mortality.

## Figures and Tables

**Table 1:** Baseline characteristics of recipients.

Recipient’s Characteristics	SD (n = 140) N (%) or Median ± IQR	ECD (n = 96) N (%) or Median ± IQR	*p*-Value
Age (years)	59 (51.5–64)	64 (58–68)	**0.000**
Female sex	22 (16%)	20 (21%)	0.312
BSA (m^2^)	1.82 (±0.20)	1.81 (±0.20)	0.917
Total Bilirubin (mg/dL)	0.95 (0.67–1.51)	1.02 (0.78–1.60)	0.272
C- Reactive Protein (mg/L)	6.3 (4.00–21.75)	5.00 (13.4–22.20)	0.061
eGFR (mL/min/1.73 m^2^)	60.0 (46.0–79.5)	55.5 (40.50–77.00)	0.196
Hypertension	83 (59%)	62 (65%)	0.411
PVD	34 (24%)	24 (25%)	0.900
COPD	51 (36%)	29 (30%)	0.321
Diabetes Mellitus	28 (20%)	22 (23%)	0.590
Previous Cardiac Surgery	45 (32%)	28 (30%)	0.627
Admission Diagnosis			
Dilatative	54 (39%)	37 (39%)	0.996
Ischemic	56 (40%)	36 (37%)	0.699
Other	30 (21%)	23 (24%)	0.407
Waitlist status			0.190
National Emergency	37 (26%)	21 (22%)	
Macro-area Emergency	49 (35%)	29 (30%)	
VA-ECMO	39 (27%)	21 (21%)	0.300
CRRT	15 (11%)	2 (2%)	**0.012**
Mechanical Ventilation	20 (14%)	3 (3%)	**0.005**

Baseline characteristics of heart transplant recipients. Continuous variables are presented as mean ± standard deviation (SD) or median (interquartile range, IQR), as appropriate. Categorical variables are expressed as a number (percentage), N (%). Abbreviations: BSA, body surface area; COPD, chronic obstructive pulmonary disease; CRRT, continuous renal replacement therapy; ECD, extended criteria donor; eGFR, estimated glomerular filtration rate; SD: standard donor; PVD, peripheral-vascular disease; VA-ECMO, veno-arterial extracorporeal membrane oxygenation. Bold values indicate statistically significant results (*p* < 0.05).

**Table 2:** Baseline donor characteristics.

Variables	SD (n = 140) N (%) or Median (IQR)	ECD (n = 96) N (%) or Median (IQR)	*p*-Value
Age (years)	47 (37.0–54.0)	65 (62.0–69.5)	**0.000**
Ischemic time (minutes)	220.0 (190.0–240.0)	218.5 (190.0–240.0)	0.611
F donor/M recipient	45 (32%)	25 (26%)	0.313
OHCA	31 (22%)	12 (12%)	0.059
On Inotropes	70 (50%)	60 (62%)	0.058
Dyslipidemia	56 (40%)	65(68%)	**0.000**
Hypertension	51 (36%)	64 (67%)	**0.000**
Diabetes Mellitus	13 (9%)	20 (21%)	**0.012**

OHCA: out-of-hospital cardiac arrest. Bold values indicate statistically significant results (*p* < 0.05).

**Table 3:** Heart transplant post-operative outcomes.

Post-Operative Outcomes	SD (n = 140) N (%) or Median ± IQR	ECD (n = 96) N (%) or Median ± IQR	*p*-Value
VA-ECMO	56 (40%)	45 (47%)	0.294
CRRT	63 (45%)	39 (40%)	0.505
CVE	4 (3%)	0 (0.0%)	0.095
Sepsis	54 (39%)	35 (36%)	0.742
In-Hospital Mortality	28 (20%)	21 (22%)	0.727
30-day Mortality	18 (13%)	10 (10%)	0.569
One-year Mortality	36 (26%)	27 (28%)	0.681
Mortality during the whole follow-up	37 (26%)	31 (32%)	0.329
Follow-up Time (months)	8.66 (1.66–21.04)	5.70 (1.22–17.18)	0.132

CRRT: continuous renal replacement therapy; CVE: cerebrovascular event; VA-ECMO: extracorporeal membrane oxygenation.

**Table 4:** Results of our univariate Cox regression model for all-cause mortality during the study period.

Recipient Variables	HR (95% CI) Univariate	*p*-Value
Age (years)	1.02 (0.99–1.05)	0.128
Bilirubin	1.17 (1.01–1.36)	**0.036**
CRP	1 (1–1.01)	**0.008**
eGFR	0.99 (0.98–1)	0.243
Length of pre-Htx ICU stay (days)	1.03 (1–1.05)	0.053
Ischemic Time (min)	1 (1–1.01)	**0.031**
Female	1.48 (0.83–2.62)	0.184
Genetic Etiology	0.91 (0.51–1.63)	0.752
Dyslipidemia	1.14 (0.68–1.93)	0.621
Hypertension	0.92 (0.56–1.49)	0.723
Cancer	1.21 (0.58–2.53)	0.614
Diabetes Mellitus	1.64 (0.97–2.77)	0.063
PVD	1.91 (1.16–3.15)	**0.011**
COPD	1.72 (1.07–2.79)	**0.026**
ICD	0.7 (0.43–1.14)	0.156
Previous Stroke or TIA	0.88 (0.51–1.52)	0.651
Redo Cardiac Surgery	1.69 (1.03–2.76)	**0.036**
VAD pre-HTx	1.18 (0.16–8.49)	0.873
Pre-op ECMO	2.59 (1.59–4.21)	0.000
Pre-op Inotropes	1.41 (0.87–2.3)	0.163
Pre-op MV	3.71 (2.05–6.71)	**<0.001**
Pre-op CVVH	2.62 (1.3–5.3)	**0.007**
**Donor Variables**		
Age (years)	1 (0.99–1.02)	0.894
Age Mismatch D/R	1.01 (1–1.03)	0.174
Female Sex	1.59 (0.98–2.56)	0.059
F Donor/M Recipient	1.41 (0.85–2.34)	0.178
Sex mismatch	1.49 (0.92–2.41)	0.104
OHCA	1.02 (0.55–1.86)	0.961
DCD	1.51 (0.37–6.2)	0.566
Inotropes	1.13 (0.7–1.83)	0.625
CAD	0.85 (0.31–2.33)	0.747
Dyslipidemia	1.05 (0.65–1.7)	0.842
Hypertension	0.86 (0.53–1.39)	0.536
Benign Neoplasia	0.78 (0.28–2.15)	0.631
Diabetes Mellitus	2.28 (1.32–3.96)	**0.003**
Sepsis	1.52 (0.88–2.64)	0.135
**Post-operative Outcomes**		
Rejection	1.95 (1.13–3.36)	**0.016**
ECMO	2.92 (1.78–4.77)	**<0.001**
CVVH	3.8 (2.23–6.48)	**<0.001**
Stroke/TIA	3.71 (1.16–11.88)	**0.027**
Sepsis	2.99 (1.83–4.9)	**<0.001**
PGD	3.34 (2.04–5.49)	**<0.001**
Intubation Time (hours)	1 (1–1)	**0.002**

CAD: coronary artery disease; COPD: chronic obstructive pulmonary disease; CRP: C-reactive protein; CVVH: continuous veno-venous hemofiltration; DCD: donation after cardiovascular death; ECMO: extracorporeal membrane oxygenation; eGFR: glomerular filtration; ICD: implantable cardiac defibrillator; MV: mechanical ventilation; PGD: primary graft dysfunction; PVD: peripheral vascular disease; VAD: ventricular assist device. Bold values indicate statistically significant results (*p* < 0.05).

**Table 5:** Results of our multivariate Cox regression model for all-cause mortality during the study period.

Variable	HR (95% CI) Multivariate	*p*-Value
Extended criteria	1.44 (0.85–2.44)	0.176
Age (years)	1.01 (0.99–1.04)	0.152
Ischemic Time (min)	1 (1–1.01)	**0.022**
PVD	1.81 (1.09–3.00)	**0.011**
COPD	1.81 (1.09–3)	**0.022**
MV pre-HTx	5.11 (2.70–9.69)	**<0.001**

MV: mechanical ventilation; COPD: chronic obstructive pulmonary disease; Bold values indicate statistically significant results (*p* < 0.05).

## Data Availability

The original contributions presented in this study are included in the article. Further inquiries can be directed to the corresponding author.
